# Volume-Law Entropy as a Mesoscopic Anomaly

**DOI:** 10.3390/e28040444

**Published:** 2026-04-14

**Authors:** Lamine Bougueroua

**Affiliations:** Independent Researcher, London KT6 6DY, UK; lamineb@gmail.com

**Keywords:** entropy scaling, area law, volume law, Fisher information, coarse-grained thermodynamics, thermodynamic stability, entanglement entropy, gravitational thermodynamics, holographic principle

## Abstract

Area-law entropy appears in local quantum ground states, low-temperature Gibbs states, and gravitational physics, whereas classical thermodynamics is formulated with volume-extensive entropy. We propose a coarse-grained information-theoretic framework, based on an effective free-energy functional combining Fisher information, a potential term, and Shannon entropy, that organises these different scalings within a single thermodynamic picture. Comparing localisation costs, external stabilisation, and gravitational self-interaction at the level of scaling reveals three regimes. At microscopic scales, locality and low-temperature coherence enforce area-type entropy scaling. At intermediate scales, volume-law entropy emerges as an effective regime sustained by non-gravitational confinement or external support; in the absence of such support, volume-extensive entropy does not by itself define an intrinsically stable equilibrium. At large scales dominated by gravitational self-interaction, a reduced scaling analysis identifies area-type behaviour as the distinguished infrared scaling, consistent with black-hole thermodynamics and with macroscopic universality requirements. The framework clarifies the limited domain of classical extensivity and offers a unified coarse-grained perspective on the recurrence of area-law scaling across quantum and gravitational settings.

## 1. Introduction

Entropy scaling exhibits a striking and persistent dichotomy across physical regimes. Ground states of local quantum many-body systems obey area-law entanglement entropy [1,2,3], while black holes possess entropy proportional to the area of their event horizons [4,5]. In contrast, the entropy of ordinary macroscopic matter in classical thermodynamics is extensive, scaling with system volume. This coexistence of area-law and volume-law entropy across different domains of physics raises a fundamental conceptual question: why does the same quantity—entropy—exhibit radically different scaling behaviours, and under what conditions is each scaling thermodynamically consistent?

Area-law entropy plays a central role in gravitational physics. In black-hole thermodynamics, it underpins the Bekenstein–Hawking formula, while in semiclassical gravity it is closely tied to the universality of gravitational coupling. Thermodynamic and entropic approaches to gravity, notably those developed by Jacobson [6,7], Verlinde [8], and Padmanabhan [9], take area scaling as a foundational input. Similarly, holographic entropy bounds [10,11] presuppose that the maximum entropy contained in a region grows no faster than its boundary area. In quantum many-body physics, area-law entanglement has been rigorously established for large classes of local Hamiltonians and gapped ground states, and is widely understood as a consequence of locality and finite correlation length.

Despite its ubiquity, area-law entropy is typically assumed rather than derived in both gravitational and quantum-information contexts. Classical thermodynamics, on the other hand, treats volume extensivity as fundamental, with little attention paid to its regime of validity. This asymmetry obscures a key issue: whether volume-law entropy represents a truly fundamental principle, or merely an effective description valid under restrictive physical conditions. If gravity is to admit a consistent thermodynamic formulation, the entropy scaling appropriate to self-gravitating systems cannot be left as an independent postulate.

In this work, we propose a more limited but more transparent interpretation. Volume-law entropy is treated not as the default fundamental scaling of matter, but as an effective mesoscopic regime that becomes viable when quantum coherence has been lost and non-gravitational forces fix the system size. The analysis is carried out within a coarse-grained Fisher–Shannon free-energy framework, where localisation cost, potential energy, and entropy can be compared on common thermodynamic grounds.

The specific contribution of the paper is threefold. First, it formulates a single effective framework in which quantum, mesoscopic, and gravitational entropy scalings can be discussed at the level of scaling and stability. Second, it interprets classical volume extensivity as externally sustained rather than intrinsically self-stabilising. Third, under explicit infrared assumptions relevant to self-gravitating systems, it identifies area-type scaling as the distinguished macroscopic behaviour. These claims are conditional on the stated framework and do not constitute a microscopic derivation from first-principles statistical mechanics.

The analysis therefore unifies three regimes within a single thermodynamic picture: (i) a quantum coherent regime in which boundary-dominated entropy is physically natural; (ii) a mesoscopic regime in which volume-law entropy emerges as an effective, externally stabilised approximation; and (iii) a gravitational regime in which infrared consistency considerations favour a return to area-type scaling. The aim is not to replace microscopic theories of entanglement or gravity, but to clarify the domain of validity of classical extensivity and the recurrence of area-law behaviour across scales.

The structure of the paper is as follows: In Section 2, we introduce the information-theoretic free-energy functional and justify its form from principles of probabilistic inference and information geometry. Section 3 analyses the quantum regime and the breakdown of volume extensivity beyond a coherence scale. In Section 4, we show how volume-law entropy arises as a metastable mesoscopic phenomenon sustained by external stabilisation. Section 5 presents the reduced self-gravitating scaling analysis. Finally, Section 6 discusses the implications for black-hole entropy, holography, and thermodynamic approaches to gravity, and clarifies the regime of validity of classical extensivity.

## 2. Information-Theoretic Free-Energy Framework

To analyse entropy scaling across quantum, mesoscopic, and gravitational regimes within a unified setting, we adopt an information-theoretic formulation of thermodynamic free energy. The framework is effective and deliberately coarse-grained: it draws on standard principles of probabilistic inference, locality, and thermodynamic stability, without introducing microscopic model assumptions or gravitational field equations.

### 2.1. Free-Energy Functional

We consider probability densities ρ(x) defined on a spatial domain $\Omega \subset R^{3}$, normalised as

(1) $$\int_{\Omega }\rho \left(x\right)\;d^{3}x=1.$$

The thermodynamic state of the system is characterised by a Helmholtz-type free-energy functional of the form

(2) $$F\left[\rho \right]=\alpha \;I_{F}\left[\rho \right]+{\langle V\rangle }_{\rho }-\tau \;S\left[\rho \right],$$

where

(3) $$I_{F}\left[\rho \right]=\int_{\Omega }\frac{{\left|\nabla \rho \right|}^{2}}{\rho }\;d^{3}x$$

is the Fisher information, ${\langle V\rangle }_{\rho }$ denotes the expectation value of the potential energy, and S[ρ] is the thermodynamic entropy.

Functionals of the form (2) appear in information-theoretic derivations of quantum mechanics and in gradient-corrected density-functional settings [12,13,14,15]. In the present paper, Equation (2) is used as an effective coarse-grained variational ansatz rather than as a microscopic partition function derivation. To connect this ansatz with standard thermodynamic language, define the effective internal energy contribution

(4) $$U_{\mathrm{eff}}\left[\rho \right]=\alpha \;I_{F}\left[\rho \right]+{\langle V\rangle }_{\rho }.$$

Then, $F\left[\rho \right]=U_{\mathrm{eff}}\left[\rho \right]-\tau \;S\left[\rho \right]$ is the isothermal free energy, and the stationarity condition δF=0 expresses the usual balance between energetic localisation and entropic delocalisation at fixed τ.

The coefficient α sets the energetic cost of spatial localisation. For non-relativistic quantum systems, consistency with the Schrödinger equation fixes

(5) $$\alpha =\frac{{ћ}^{2}}{8m},$$

as established in information-theoretic derivations of quantum mechanics [12,13]. The parameter τ plays the role of an effective temperature controlling the balance between energetic localisation and entropic delocalisation. Throughout this work, τ is treated as an externally fixed thermodynamic parameter, appropriate to coarse-grained equilibrium descriptions.

Equation (2) should be understood as an effective free energy governing the equilibrium configuration of ρ under macroscopic constraints. It does not assume a microscopic Hamiltonian, nor does it presuppose any specific gravitational dynamics. Instead, it encodes three general physical contributions: localisation, interaction energy, and entropy.

Throughout, admissible densities satisfy ρ≥0, $\int_{\Omega }\rho \;d^{3}x=1$, and $\sqrt{\rho }\in H^{1}\left(\Omega \right)$, so that

(6) $$I_{F}\left[\rho \right]=4\int_{\Omega }{\left|\nabla \sqrt{\rho }\right|}^{2}\;d^{3}x<\infty .$$

When variational derivatives are taken, we assume either periodic boundary conditions or vanishing normal derivative on ∂Ω, so that the boundary term arising from integration by parts vanishes.

### 2.2. Why Shannon Entropy and Fisher Information Are Natural in the Present Framework

The functional form of Equation (2) is motivated by standard results in inference and information geometry, but it is not claimed to be the unique microscopic free energy of all many-body or gravitational systems. Within probabilistic updating problems, the Shore–Johnson axioms single out Shannon entropy as the distinguished additive entropy functional [16]. Likewise, within information geometry, Chentsov’s theorem singles out Fisher information as the canonical Riemannian metric on statistical manifolds [17,18]. These results explain why Shannon entropy and Fisher information are natural ingredients of a coarse-grained variational ansatz.

Accordingly, Equation (2) is used here as an effective Fisher–Shannon free-energy functional. Its role is to provide a compact arena in which localisation cost, external support, and entropy scaling can be compared at the level of thermodynamic consistency. The manuscript does not claim that Equation (2) is the only possible mesoscopic ansatz, nor that it follows directly from a microscopic partition function.

### 2.3. Locality and Scaling Behaviour

A key feature of the Fisher information term is its sensitivity to spatial gradients. For self-similar nonuniform density families of the form

(7) $$\rho_{L}\left(x\right)=L^{-3}\;f\left(x/L\right),$$

one has

(8) $$I_{F}\left[\rho_{L}\right]=L^{-2}\;I_{F}\left[f\right].$$

This is the scaling class used in the present analysis. Uniform densities, boundary-layer profiles, or multiscale configurations need not obey Equation (8), and we do not treat $I_{F}∼L^{-2}$ as a completely general statement about arbitrary densities.

For $\sqrt{\rho }\in H^{1}\left(\Omega \right)$ on a convex domain Ω with periodic or Neumann boundary conditions, the Poincaré–Wirtinger inequality [19] implies

(9) $$I_{F}\left[\rho \right]\geq \frac{4\pi^{2}}{L^{2}}\int_{\Omega }{\left(\sqrt{\rho }-\langle \sqrt{\rho }\rangle \right)}^{2}d^{3}x.$$

Equation (9) is used here only as a lower-bound illustration of inverse-length localisation cost. It does not by itself establish a universal scaling law for arbitrary densities.

By contrast, the entropy term scales with the effective number of accessible degrees of freedom. If entropy scales as

(10) $$S∼\sigma \;L^{\gamma },$$

the exponent γ characterises the extensivity of the thermodynamic description. Classical thermodynamics corresponds to γ=3, while area-law entropy corresponds to γ=2.

**Figure 1:**
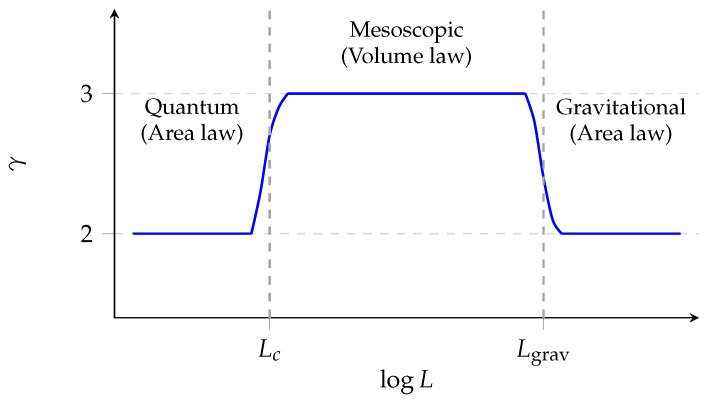
Entropy scaling exponent γ (where $S∼L^{\gamma }$) as a function of system size. Area-law scaling (γ=2) prevails at both quantum ($L≃L_{c}$) and gravitational ($L\gg L_{\mathrm{grav}}$) scales; volume-law (γ=3) is a mesoscopic anomaly sustained by external stabilisation.

As we will show, the effective value of γ depends on the physical regime, yielding a characteristic “sandwich structure” (Figure 1).

The free energy density $f=F/L^{3}$ therefore depends sensitively on the relative scaling of localisation, interaction energy, and entropy. As we show in subsequent sections, this scaling competition determines whether a thermodynamically stable equilibrium exists and whether the resulting equilibrium is universal or composition-dependent.

### 2.4. Regime of Validity and Assumptions

Several assumptions underlie the framework and delimit its regime of applicability. First, the description is coarse-grained: ρ(x) represents a mesoscopic probability density rather than a microscopic quantum state. Second, the analysis is non-relativistic in form; relativistic effects enter only through the requirement of diffeomorphism-invariant entropy functionals at macroscopic scales. Third, the framework assumes equilibrium or quasi-equilibrium configurations, allowing the use of a free-energy variational principle.

Within these limitations, Equation (2) provides a unified description of quantum localisation, thermodynamic entropy, and gravitational self-interaction. Crucially, it allows entropy scaling to be treated as a dynamical and thermodynamic question rather than an external postulate.

For mathematical completeness, Appendix A derives the stationary Euler–Lagrange equation associated with the free-energy functional, explicitly recovering the standard quantum and classical limits. Additionally, Appendix B provides a scaling consistency argument checking that, under standard infrared inputs (L∝M and $T\propto L^{-1}$), area-law entropy (γ=2) is the scaling compatible with composition-independent macroscopic geometry in the reduced description.

In the following sections, we apply this framework to three distinct regimes. We first analyse the quantum regime, where the Fisher information term dominates and enforces entanglement locality. We then show how volume-law entropy arises as an effective, externally sustained mesoscopic approximation. Finally, we examine the gravitational regime within the reduced scaling model.

## 3. Quantum Regime and the Breakdown of Volume Extensivity

We first consider the regime in which the localisation term in the free-energy functional dominates the thermodynamic balance. This regime corresponds to sufficiently small length scales, where quantum coherence is maintained and the equilibrium structure of ρ(x) is strongly constrained by locality.

### 3.1. Fisher Dominance and the Coherence Scale

At small system sizes, the Fisher information term in Equation (2) provides the leading contribution to the free energy. Using the scaling property (8) for self-similar densities, the Fisher contribution scales as $L^{-2}$, with a lower bound supported by Equation (9).

The competition between localisation and entropy defines a crossover scale at which the two contributions become comparable at the level of order of magnitude. Using Equation (8) together with $\tau =k_{B}T$, one obtains

(11) $$L_{c}∼\sqrt{\frac{{ћ}^{2}}{8mk_{B}T}},$$

up to profile-dependent factors. The quantity $L_{c}$ should be read as a parametric crossover scale rather than a profile-independent exact derivation from Equation (2). Its role is to mark the regime in which localisation costs and entropic delocalisation become comparable within the effective description.

The scale $L_{c}$ thus marks the boundary between a quantum-coherent regime and a decohered, thermodynamically classical regime. Its magnitude is consistent with experimentally observed decoherence thresholds in matter-wave interferometry and macromolecular superposition experiments.

### 3.2. Entanglement Locality and Area-Law Entropy

In the regime $L≲L_{c}$, local quantum systems in ground states, gapped phases, or sufficiently low-temperature Gibbs states are known to exhibit area-law entanglement under appropriate locality conditions. This entanglement entropy should not be identified literally with the coarse-grained Shannon entropy entering Equation (2). Rather, entanglement provides the physical motivation for the ultraviolet area-law regime, while the Fisher–Shannon functional provides an effective thermodynamic language for organising that regime.

Lieb–Robinson bounds are used here only to motivate the locality structure underlying area-law behaviour in local Hamiltonians [20,21]; they are not presented as a standalone derivation of equilibrium entropy scaling. In this setting, the entanglement between a spatial region *A* and its complement is boundary dominated and scales as

(12) $$S_{\mathrm{ent}}\left(A\right)\leq c\;\left|\partial A\right|.$$

Area-law entanglement has been established in broad classes of local quantum systems and related settings [1,22,23,24,25,26,27,28].

This discussion does not extend to generic highly excited eigenstates. In nonintegrable systems satisfying the eigenstate thermalisation hypothesis (ETH), highly excited states typically exhibit volume-law entanglement, and this should be kept conceptually distinct from the coarse-grained thermodynamic volume law discussed later in the paper. The present claim is therefore restricted to the coherence-dominated low-temperature/locality regime, not to arbitrary excited-state equilibrium physics.

Within the present framework, area-law entropy is not imposed as an axiom. Rather, in the coherence-dominated regime it emerges as a natural thermodynamic consequence of Fisher dominance combined with locality of interactions: the Fisher information term penalises rapid spatial variation in ρ, effectively suppressing bulk degrees of freedom and confining entropy production to boundary regions. Within this effective description, area-type scaling is therefore the natural outcome when quantum coherence is maintained.

**Example** **1.** *A gapped spin chain, or a trapped-ion many-body state in the appropriate low-temperature/locality-dominated regime [29,30], provides a concrete realisation of this regime: correlations are local, entanglement is boundary dominated, and the effective coarse-grained description is not bulk-extensive.*

### 3.3. Loss of Coherence and the Opening of the Mesoscopic Regime

As the system size exceeds the coherence length $L_{c}$, the Fisher contribution to the free energy becomes subdominant. Quantum coherence is progressively suppressed by thermal fluctuations and environmental coupling, and the equilibrium distribution ρ becomes effectively classical. In this regime, Shannon entropy governs the thermodynamic behaviour, and volume-extensive entropy appears to be favoured.

Importantly, the emergence of volume-law entropy at $L\gg L_{c}$ does not signal a fundamental change in the nature of entropy itself. Rather, it reflects the loss of quantum constraints that previously enforced locality of information. The system enters a regime in which entropy can, in principle, occupy the bulk.

However, as we show in the following section, this volume-extensive behaviour is not intrinsically stable. It persists only in the presence of external stabilisation mechanisms that fix the system size or energy density. In the absence of such stabilisation, volume-law entropy fails to define a self-consistent thermodynamic equilibrium.

The quantum-to-mesoscopic transition thus marks the opening of a regime in which volume-law entropy may appear, but only as an effective and externally sustained approximation. This observation sets the stage for analysing the conditions under which volume extensivity breaks down and for identifying the entropy scaling required by gravitational stability.

The mesoscopic volume law discussed below concerns coarse-grained thermodynamic entropy under external stabilisation. It should not be conflated with the volume-law entanglement of generic highly excited pure states in ETH settings, which lies outside the present equilibrium coarse-grained description.

## 4. Classical Thermodynamics as an Effective Description

We now examine the regime in which quantum coherence has been suppressed but gravitational self-interaction remains negligible. This intermediate domain corresponds to length scales

(13) $$L_{c}\ll L\ll L_{\mathrm{grav}},$$

where $L_{\mathrm{grav}}$ denotes the scale at which gravitational effects become thermodynamically significant. It is within this window that classical thermodynamics operates and volume-law entropy appears to be stable.

### 4.1. External Stabilisation and Effective Extensivity

In the absence of quantum coherence, the Fisher information term in the free energy becomes subdominant, and entropy maximization favours bulk occupation of available degrees of freedom. For ordinary laboratory systems, this leads to entropy scaling proportional to the system volume. However, such systems are invariably subject to external stabilisation mechanisms.

A paradigmatic example is the ideal gas confined within rigid container walls. The volume-extensive entropy of the gas arises only because the container imposes an external length scale that fixes the spatial domain Ω. Similarly, condensed matter systems rely on electromagnetic bonding, lattice rigidity, or externally imposed boundary conditions to maintain a finite equilibrium size. In all such cases, the energy density is effectively fixed by non-gravitational forces.

Within the present framework, these stabilisation mechanisms enter through the potential energy term ${\langle V\rangle }_{\rho }$ in Equation (2). They provide a restoring force that counteracts entropic expansion and allows a volume-extensive entropy to coexist with thermodynamic equilibrium. The resulting extensivity is therefore not intrinsic to entropy itself, but contingent on the presence of external constraints.

**Example** **2.** *An ideal gas in a rigid container and a crystal stabilised by electromagnetic bonding both realise this mesoscopic regime: the volume law is meaningful only because walls, lattice forces, or chemical bonding fix the domain size and enter through ${\langle V\rangle }_{\rho }$.*

### 4.2. Absence of Intrinsic Equilibrium for Volume-Law Entropy

The dependence of volume-law entropy on external stabilisation becomes evident when such constraints are removed. Consider a system whose entropy scales as

(14) $$S∼\sigma \;L^{3},$$

but which is otherwise free to expand. In the absence of confining forces, the entropy term in the free energy grows without bound as *L* increases, while the localisation term is negligible. The free energy therefore decreases monotonically with system size, and no finite equilibrium configuration exists.

This observation highlights a crucial distinction between entropy scaling and equilibrium existence. Volume-law entropy does not, by itself, define a stable thermodynamic state. Rather, it presupposes an externally fixed volume or energy density. Classical thermodynamics implicitly assumes such stabilisation and is therefore silent on the behaviour of truly isolated macroscopic systems.

From this perspective, volume extensivity is an effective description valid only within a restricted domain. It is sustained by non-gravitational forces that dominate over both quantum coherence and gravitational self-interaction. Once these forces are removed or rendered negligible, the volume-law description ceases to be self-consistent.

### 4.3. Mesoscopic Anomaly and Its Limits

The appearance of volume-law entropy in the intermediate regime $L_{c}\ll L\ll L_{\mathrm{grav}}$ can thus be understood as a mesoscopic anomaly. It reflects a balance in which entropy is allowed to fill the bulk because neither quantum locality nor gravitational instability imposes a stronger constraint. This balance is inherently fragile and disappears outside the mesoscopic window.

Importantly, the anomaly is not associated with any singular behaviour or phase transition. Rather, it marks a crossover between two regimes in which area-law entropy is enforced for different reasons. On small scales, the locality of information propagation confines entropy to boundaries. On large scales, as we show in the next section, thermodynamic stability and infrared consistency impose a similar constraint.

The mesoscopic regime therefore occupies a special but limited place in the hierarchy of entropy scalings. It explains the empirical success of classical thermodynamics while simultaneously clarifying its domain of validity. Volume-law entropy emerges as a contingent approximation rather than a fundamental principle, setting the stage for the analysis of gravitational systems in which no external stabilisation is available.

## 5. Gravitational Regime: Reduced Self-Gravitating Scaling Analysis

We now turn to the regime in which gravitational self-interaction becomes thermodynamically relevant. The aim of this section is limited, it is not to provide a full model of realistic self-gravitating matter with pressure support, kinetic terms, or detailed equations of state. Rather, it uses a reduced scaling model to ask which entropy scalings can plausibly remain compatible with an intrinsic macroscopic description once external stabilisation is removed.

### 5.1. Reduced Self-Gravitating Model

Consider a system of total mass *M* and characteristic size *L*, with effective entropy scaling

(15) $$S_{\mathrm{eff}}\left(L\right)∼\sigma \;L^{\gamma }.$$

For a self-gravitating configuration, the dominant attractive contribution to the potential energy scales as

(16) $${\langle V\rangle }_{\rho }∼-\frac{GM^{2}}{L},$$

up to geometry-dependent factors. Retaining only this contribution together with the entropy term, the reduced free energy becomes

(17) $$F_{\mathrm{red}}\left(L\right)≃-\frac{GM^{2}}{L}-\tau \;\sigma L^{\gamma }.$$

This expression is deliberately incomplete because it omits pressure, kinetic support, and equation-of-state physics. Its purpose is not to predict realistic hydrostatic equilibria, but to test whether the entropy term by itself can furnish intrinsic stabilisation once external support is absent.

A stationary point of Equation (17), when it exists, satisfies

(18) $$\frac{GM^{2}}{L^{2}}-\gamma \tau \sigma L^{\gamma -1}=0.$$

**Figure 2:**
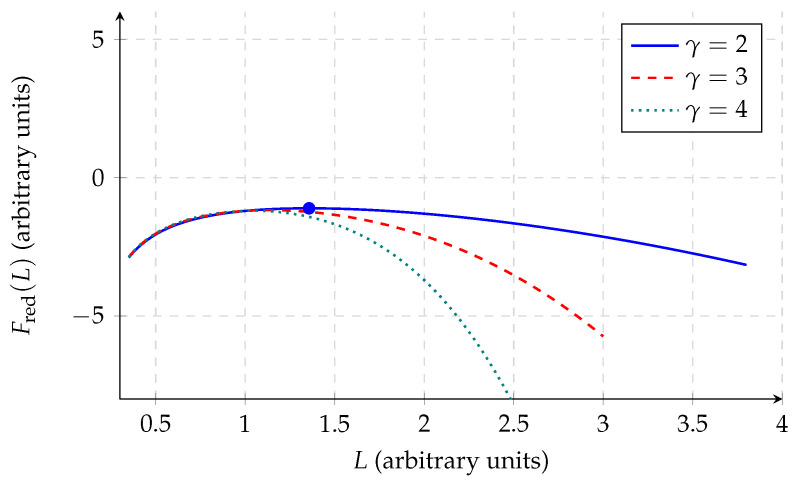
Reduced free energy $F_{\mathrm{red}}\left(L\right)$ for representative exponents γ=2,3,4 at fixed $GM^{2}=1$ and τσ=0.2. The curves illustrate that, without additional positive support terms, the reduced model does not develop an intrinsic stable minimum; any stationary point (filled circle, γ=2 case) is a local maximum due to Equation (19). The role of the reduced model is therefore diagnostic rather than hydrostatic.

However,

(19) $$\frac{d^{2}F_{\mathrm{red}}}{dL^{2}}=-\frac{2GM^{2}}{L^{3}}-\gamma \left(\gamma -1\right)\;\tau \sigma L^{\gamma -2},$$

which is negative at the stationary point for γ>1. The reduced model therefore does not generate a stable minimum. Its implication is correspondingly narrower: bulk entropy alone is not an intrinsic stabilising mechanism for isolated self-gravitating matter. Any realistic equilibrium requires additional positive support terms, whose detailed treatment lies outside the present coarse-grained analysis (Figure 2).

### 5.2. Macroscopic Universality and Composition Dependence

The remaining issue is not whether Equation (17) by itself produces a realistic star or isothermal sphere—it does not—but whether different entropy scalings transmit microscopic coefficients too directly into macroscopic gravitational behaviour. Let σ denote the coefficient in $S_{\mathrm{eff}}∼\sigma L^{\gamma }$. In the present coarse-grained parametrization, σ may inherit dependence on microscopic inputs, including the kinetic coefficient

(20) $$\alpha =\frac{{ћ}^{2}}{8m}.$$

For generic γ, crossover or equilibrium relations constructed from the reduced description then carry this microscopic coefficient directly into macroscopic length scales. The concern emphasised here is therefore macroscopic universality, where useful gravitational thermodynamics should not exhibit uncontrolled sensitivity of its large-scale geometry to microscopic entropy coefficients.

### 5.3. Why Area-Type Scaling Remains Distinguished

Within the present framework, area-type scaling remains distinguished for two reasons. First, it matches the infrared scaling already exhibited by black-hole thermodynamics. Second, under the standard infrared relations L∝M and $T\propto L^{-1}$, Appendix B shows that γ=2 keeps the entropic contribution TS commensurate with gravitational self-energy without forcing σ to acquire explicit system-size dependence. For that reason, we treat γ=2 not as a universally proven microscopic theorem, but as the preferred infrared scaling within the stated coarse-grained and universality assumptions.

The conclusion of this section is therefore conditional: once external support is removed and one imposes macroscopic universality together with the infrared consistency conditions summarised in Appendix B, area-type scaling is the distinguished gravitational behaviour in the present framework.

### 5.4. The Sandwich Structure

The analysis of the preceding sections reveals a coherent hierarchy of entropy scalings, summarised in Table 1. Area-law entropy brackets volume-law from both above and below, with the intermediate mesoscopic regime sustained only by external stabilisation mechanisms.

The key insight is that volume-law entropy does not interpolate between two unrelated area-law regimes; rather, it represents a temporary suspension of the locality constraints that enforce area scaling at both extremes. The vast separation between $L_{c}$ (nanometres) and $L_{\mathrm{grav}}$ (planetary scales) for ordinary matter explains why volume-extensive thermodynamics dominates human experience, even though it is not the fundamental scaling.

### 5.5. Relation to Black-Hole Entropy

The Schwarzschild black hole is not merely an illustrative example of the infrared regime; it provides the only known physical system in which the two infrared inputs invoked in Appendix B hold *exactly*. The Schwarzschild radius is $r_{s}=2GM/c^{2}$, so L∝M is exact. The Hawking temperature is $T_{H}=ћc^{3}/\left(8\pi GMk_{B}\right)$, so $T\propto L^{-1}$ is exact. The consistency check of Appendix B therefore applies without approximation: under the assumed infrared relations, the exponent keeping σ size-independent is γ=2, and $S∼L^{2}∼r_{s}^{2}$ follows directly.

This scaling can also be seen from the first law of black-hole mechanics alone, without invoking holography or microscopic models. Given $T_{H}\propto M^{-1}$, the thermodynamic identity $dM=T_{H}\;dS$ requires

(21) $$dS\propto M\;dM\Rightarrow S\propto M^{2}\propto r_{s}^{2}\propto A,$$

where $A=4\pi r_{s}^{2}$ is the horizon area. A volume-law ansatz $S∼r_{s}^{3}∼M^{3}$ would instead give $T_{H}\;dS∼M^{-1}\cdot 3M^{2}\;dM=3M\;dM\neq dM$, violating the first law for all M>1 in natural units. The first law is therefore inconsistent with volume-law scaling, given the Hawking temperature, where within this thermodynamic argument, area-type scaling is the distinguished power-law behaviour. This is a direct thermodynamic consistency check, not merely a numerical coincidence.

The black hole also realises, in its most transparent form, the composition-independence condition of Section 5. By the no-hair theorem, $S_{BH}$ depends only on *M*, *J*, and *Q*—macroscopic invariants—and not on the microscopic constituents from which the black hole formed. This is precisely the universality property that the reduced model identifies as a constraint: the entropy coefficient σ in $S∼\sigma L^{2}$ must not carry residual microscopic dependence into macroscopic gravitational geometry. For a Schwarzschild black hole, $\sigma =k_{B}c^{3}/\left(4Gћ\right)$ contains only universal constants; there is no dependence on particle mass, species, or equation of state.

These two observations—exact realisation of the infrared scaling relations and composition-independence of $S_{BH}$—together identify the Schwarzschild solution as the infrared fixed point of the entropy-scaling picture developed here. What the present framework does *not* provide is a derivation of the numerical prefactor 1/4 in $S_{BH}=A/\left(4Gћ\right)$; recovering that the coefficient requires a microscopic theory such as string theory or loop quantum gravity, which lies outside the scope of the present coarse-grained analysis.

A further parallel with the present framework arises in recent work on the black-hole information paradox. The island formula [31,32], which resolves the Page curve problem via a gravitational replica trick, involves a genuine competition between two entropy contributions: a volume-law piece from Hawking radiation accumulating outside the black hole, and an area-law saddle-point contribution from an island region behind the horizon. The transition at the Page time—where the area-law saddle comes to dominate—maps naturally onto the crossover from mesoscopic volume-law to gravitational area-law behaviour identified here. In both cases, volume-law entropy is the initial effective description, but area-law scaling becomes dominant once gravitational degrees of freedom are correctly accounted for.

## 6. Discussion, Empirical Scales, and Conclusions

### 6.1. Empirical Scales and Physical Interpretation

Although the analysis presented in this work is purely theoretical, it predicts two physically meaningful crossover scales that delineate the regimes of entropy scaling. The first is the coherence length $L_{c}$ introduced in Equation (11), which separates quantum-coherent behaviour from classical thermodynamics. For molecular masses at room temperature, $L_{c}$ lies in the nanometre range, consistent with experimentally observed decoherence thresholds in matter-wave interferometry and macromolecular superposition experiments.

The second is the gravitational scale $L_{\mathrm{grav}}$, defined implicitly as the system size at which gravitational self-interaction becomes thermodynamically relevant. Dimensional analysis yields

(22) $$L_{\mathrm{grav}}∼{\left(\frac{k_{B}T}{G\rho^{2}}\right)}^{1/4},$$

where ρ is the characteristic mass density. For ordinary condensed matter densities, this scale lies in the range $10^{6}$–$10^{7}$ m, coinciding with the astrophysical transition at which material rigidity can no longer counteract self-gravity. This scale is closely related to the so-called “potato radius” that separates irregular bodies from approximately spherical ones in planetary science.

These crossover scales are not free parameters to be fitted, but consistency checks that support the physical interpretation of the framework. They indicate that the mesoscopic regime of volume-law entropy occupies a finite window between two asymptotic domains in which area-law scaling is enforced for independent reasons.

### 6.2. Relation to Thermodynamic and Entropic Gravity

Recent developments in quantum information provide further support for the picture presented here. Measurement-induced entanglement phase transitions, in which the entanglement scaling of a quantum state changes from volume-law to area-law as the rate of projective measurement increases, have been demonstrated both theoretically and experimentally [33,34,35]. These transitions illustrate that volume-law entanglement is fragile and can be driven to area-law scaling by local information extraction, consistent with our identification of volume-law entropy as an effective mesoscopic anomaly. In a complementary direction, area-law scaling has been established for entanglement entropy in high-energy particle scattering [36], extending the universality of area-law entropy beyond lattice systems to relativistic quantum field theory.

The present interpretation is also consistent with recent literature on area laws in thermal and long-range systems, with experimental evidence for area-law behaviour in trapped-ion many-body states [30], and with typicality/ETH-based arguments showing that generic highly excited pure states can exhibit volume-law entanglement [37,38,39]. These developments support a more limited claim than that made in the original manuscript: area-type scaling is natural in low-temperature/locality-dominated and self-gravitating infrared regimes, whereas volume-law behaviour belongs either to externally sustained thermodynamic descriptions or to highly excited pure-state entanglement settings outside the present coarse-grained equilibrium framework.

The present results are consistent with thermodynamic approaches to gravity. In Jacobson’s derivation of the Einstein equations as an equation of state [6,7], area-law entropy is assumed as an input. Within the present framework, this area-law input is consistent with the infrared scaling requirements identified here; alternative entropy scalings would not satisfy the reduced model’s universality condition under the same infrared assumptions.

Similarly, entropic force models of gravity implicitly rely on area scaling to ensure universality of the emergent force. The present coarse-grained perspective supports this reliance, independent of holographic dualities or microscopic spacetime models; within the reduced self-gravitating framework, the conclusion extends to self-gravitating systems admitting an equilibrium description, regardless of whether horizons are present.

The framework also complements holographic entropy bounds. Rather than postulating an upper bound on entropy, it explains why area-type scaling is the distinguished infrared behaviour in regimes where gravitational self-interaction dominates within the present coarse-grained picture. From this perspective, holographic bounds reflect a thermodynamic consistency pattern rather than a distinct principle.

### 6.3. Scope and Limitations

Several limitations of the present analysis should be emphasised. First, the framework is coarse-grained and equilibrium-based; it does not address far-from-equilibrium dynamics or strongly time-dependent gravitational systems. Second, the analysis focuses on entropy scaling rather than absolute normalisation. While it clarifies why area-type scaling is distinguished, it does not derive the numerical coefficient appearing in the Bekenstein–Hawking formula. Third, the treatment is non-relativistic in form, relying on scaling arguments rather than a full covariant field-theoretic description. Fourth, the reduced gravitational model in Section 5 omits pressure, kinetic support, and realistic equations of state; its conclusions are diagnostic rather than hydrostatic.

These limitations are deliberate. The goal of this work is not to provide a microscopic theory of gravity, but to identify thermodynamic consistency conditions within a coarse-grained framework. Within this scope, the results are conditional on the stated assumptions and provide a coherent organisational picture of entropy scaling across regimes.

### 6.4. Conclusions

We have argued, within a coarse-grained Fisher–Shannon framework, that volume-law entropy is best understood as an effective feature of a mesoscopic regime sustained by external stabilisation. The analysis does not constitute a microscopic derivation from first-principles statistical mechanics. Its claim is more limited: once localisation costs, external support, and gravitational self-interaction are compared at the level of scaling, the domain of validity of classical extensivity becomes sharply restricted.

Within this framework, area-type scaling is the natural boundary-dominated behaviour in ground-state and low-temperature local quantum systems and the distinguished infrared behaviour in the self-gravitating regime, while volume-law behaviour occupies an intermediate thermodynamic window. This picture is consistent with recent experimental demonstrations of area-law entanglement in trapped-ion quantum simulators [29,30] and with typicality arguments showing that highly excited pure states generically exhibit volume-law entanglement [37,38,39]. In this sense, the recurrence of area-law entropy across disparate contexts is interpreted here as a common coarse-grained consistency pattern rather than as evidence for identical microscopic mechanisms.

By identifying entropy scaling as a consistency condition rather than an independent postulate, the present work organises the appearance of area-law entropy in quantum entanglement, black-hole thermodynamics, and gravitational universality within a single coarse-grained picture. It clarifies the domain of validity of classical extensivity and constrains thermodynamic approaches to gravity, holography, and emergent spacetime. The recurrence of area-law entropy across disparate physical contexts thus reflects a shared stability principle at the level of the present effective framework.

## Figures and Tables

**Table 1:** Entropy scaling across physical regimes. Area-type scaling is the natural behaviour at both ultraviolet (quantum) and infrared (gravitational) limits within the present framework; volume-law is the intermediate anomaly.

Regime	Scale	Scaling	Mechanism
Quantum	$L≲L_{c}$	S∼A	Entanglement locality
Mesoscopic	$L_{c}\ll L\ll L_{\mathrm{grav}}$	S∼V	External stabilisation
Gravitational	$L\gg L_{\mathrm{grav}}$	S∼A	Infrared consistency

## Data Availability

This work is purely theoretical; no data were generated or analysed.

## Appendix A. Variational Derivation of the Stationarity Equation

The derivation in this appendix assumes the admissibility and boundary conditions stated in Section 2, so that the boundary term generated by integration by parts vanishes.

We derive the equilibrium condition for ρ(x) by minimising F[ρ] defined in Equation (2), subject to $\int \rho \;d^{3}x=1$. We introduce the real amplitude ψ(x) such that $\rho \left(x\right)=\psi^{2}\left(x\right)$. In terms of ψ:

(A1) $$I_{F}\left[\rho \right]=4\int_{\Omega }{\left|\nabla \psi \right|}^{2}\;d^{3}x,$$

(A2) $$S\left[\rho \right]=-2\int_{\Omega }\psi^{2}\ln\left|\psi \right|\;d^{3}x.$$

With a Lagrange multiplier μ enforcing normalisation,

(A3) $$L\left[\psi \right]=4\alpha \;\int \;{\left|\nabla \psi \right|}^{2}\;d^{3}x+\int \;V\psi^{2}\;d^{3}x+2\tau \;\int \;\psi^{2}\ln\left|\psi \right|\;d^{3}x-\mu \;\left(\int \;\psi^{2}\;d^{3}x-1\right).$$

Setting δL/δψ=0 and integrating by parts yields

(A4) $$-8\alpha \nabla^{2}\psi +2V\psi +2\tau \left(2\psi \ln|\psi |+\psi \right)-2\mu \psi =0.$$

Dividing by 2 and absorbing the linear term into the multiplier ($\tilde{\mu }=\mu -\tau$) gives the stationary equation

(A5) $$-4\alpha \nabla^{2}\psi +V\left(x\right)\psi +2\tau \psi \ln\left|\psi \right|=\tilde{\mu }\psi .$$

Equation (A5) is a nonlinear elliptic equation of logarithmic Schrödinger type. For fixed external V(x), the Fisher term $I_{F}\left[\rho \right]$ and the contribution −τS[ρ] are convex on the admissible set of positive normalised densities, while ${\langle V\rangle }_{\rho }$ is affine. Accordingly, the free-energy functional is convex in the fixed-potential setting considered in this appendix. This statement need not persist in reduced self-gravitating models where the effective potential is itself configuration dependent.

Equation (A5) interpolates between two fundamental regimes:

1. **Quantum Regime** (τ→0): The logarithmic term vanishes. Setting $\alpha ={ћ}^{2}/8m$ recovers the time-independent Schrödinger equation Hψ=Eψ with $E=\tilde{\mu }$.
2. **Classical Regime** (α→0): The gradient term vanishes. The equation reduces to $V+\tau \ln(\psi^{2})=\mathrm{const}$, yielding the Boltzmann distribution ρ∝exp(−V/τ).

## Appendix B. Infrared Scaling Consistency Check

This appendix does not provide an independent derivation of γ=2. Its purpose is narrower: assuming the infrared relations L∝M and $T\propto L^{-1}$ appropriate to relativistic self-gravitating systems, it checks which entropy exponent keeps the entropic contribution TS commensurate with gravitational self-energy without introducing explicit system-size dependence into the coefficient σ.

We assume $S∼\sigma L^{\gamma }$ with σ a microscopic constant, and invoke:

1. **Geometric universality:** L∝M (e.g., Schwarzschild radius $r_{s}=2GM/c^{2}$).
2. **Gravitational temperature:** $T\propto L^{-1}$ (Hawking/Unruh scalings).

Comparing scaling dimensions (using M∼L):

(A6) $$\begin{array}{cc} E_{\mathrm{grav}} & ∼\frac{M^{2}}{L}∼L, \end{array}$$

(A7) $$\begin{array}{cc} E_{\mathrm{entropic}} & ∼TS∼\left(L^{-1}\right)\cdot \left(\sigma L^{\gamma }\right)∼\sigma L^{\gamma -1}. \end{array}$$

Commensurability requires equal exponents:

(A8) 1=γ−1⇒γ=2.

This identifies area-type scaling (γ=2) as the exponent consistent with the assumed infrared relations while keeping σ as a fixed microscopic coefficient. Conversely, volume-law entropy (γ=3) would require $\sigma ∼L^{-1}$, a system-size-dependent coefficient, violating the assumption that σ is a fixed microscopic parameter. This supports the use of area-type scaling in the infrared discussion of the main text and should be read as a consistency check conditional on the assumed infrared relations.
